# Using High-Throughput Sequencing to Leverage Surveillance of Genetic Diversity and Oseltamivir Resistance: A Pilot Study during the 2009 Influenza A(H1N1) Pandemic

**DOI:** 10.1371/journal.pone.0067010

**Published:** 2013-07-02

**Authors:** Juan Téllez-Sosa, Mario Henry Rodríguez, Rosa E. Gómez-Barreto, Humberto Valdovinos-Torres, Ana Cecilia Hidalgo, Pablo Cruz-Hervert, René Santos Luna, Erik Carrillo-Valenzo, Celso Ramos, Lourdes García-García, Jesús Martínez-Barnetche

**Affiliations:** 1 Centro de Investigaciones sobre Enfermedades Infecciosas, Instituto Nacional de Salud Pública, Cuernavaca, México; 2 Centro de Información para Decisiones en Salud Pública, Instituto Nacional de Salud Pública, Cuernavaca, México; 3 Departamento de Epidemiología, Servicios de Salud de Morelos, Cuernavaca, México; The University of Hong Kong, Hong Kong

## Abstract

**Background:**

Influenza viruses display a high mutation rate and complex evolutionary patterns. Next-generation sequencing (NGS) has been widely used for qualitative and semi-quantitative assessment of genetic diversity in complex biological samples. The “deep sequencing” approach, enabled by the enormous throughput of current NGS platforms, allows the identification of rare genetic viral variants in targeted genetic regions, but is usually limited to a small number of samples.

**Methodology and Principal Findings:**

We designed a proof-of-principle study to test whether redistributing sequencing throughput from a high depth-small sample number towards a low depth-large sample number approach is feasible and contributes to influenza epidemiological surveillance. Using 454-Roche sequencing, we sequenced at a rather low depth, a 307 bp amplicon of the neuraminidase gene of the Influenza A(H1N1) pandemic (A(H1N1)pdm) virus from cDNA amplicons pooled in 48 barcoded libraries obtained from nasal swab samples of infected patients (n  =  299) taken from May to November, 2009 pandemic period in Mexico. This approach revealed that during the transition from the first (May-July) to second wave (September-November) of the pandemic, the initial genetic variants were replaced by the N248D mutation in the NA gene, and enabled the establishment of temporal and geographic associations with genetic diversity and the identification of mutations associated with oseltamivir resistance.

**Conclusions:**

NGS sequencing of a short amplicon from the NA gene at low sequencing depth allowed genetic screening of a large number of samples, providing insights to viral genetic diversity dynamics and the identification of genetic variants associated with oseltamivir resistance. Further research is needed to explain the observed replacement of the genetic variants seen during the second wave. As sequencing throughput rises and library multiplexing and automation improves, we foresee that the approach presented here can be scaled up for global genetic surveillance of influenza and other infectious diseases.

## Introduction

The first cases of a new H1N1 Influenza A virus derived from swine, human and avian triple reassortant Influenza A viruses were detected in April 2009 in Mexico and the United States [1]. The rapid spread of influenza cases that occurred in subsequent months led World Health Organization to raise the pandemic alert to level 6; this being the first pandemic of the 21st century. During the first year of the pandemic, 18,500 deaths occurred as a consequence of laboratory confirmed A(H1N1)pdm infection. However, recent estimates point out that the death toll may be 15 times higher, mainly due to underreporting in developing countries [2].

Clinical management is important for reducing mortality and the use of antivirals is of great value to limit infection with influenza virus. In the first weeks of the 2009 pandemic, it was determined that the new strain was resistant to adamantanes, but sensitive to the neuraminidase (NA) inhibitors oseltamivir (Ose) and zanamivir (Zan). These drugs represented an important therapeutic tool as prophylactic agents and for management of clinical infections [3].

Similar to other ssRNA viruses, influenza viruses have a high mutation rate, allowing antigenic variation (drift) and the consequent seasonal limitations on the use of vaccines for control [4]. In addition, co-infection with different influenza viruses can lead to genome segment reassorment causing the emergence of novel antigenic “shifted” viruses such as the one causing the 2009 pandemic. Certain clinically relevant NA gene mutations are strongly associated with phenotypic resistance to Ose (H275Y and I223R) and Zan (Q136K,) in seasonal Influenza A and A(H1N1)pdm viruses [3], [5]. In the United States of America before the 2007–2008 season, the prevalence of Ose resistant seasonal influenza A strains was less than 1%. However a rapid increase was observed in the following seasons such that in the pre-pandemic season, 99.4% of seasonal H1N1 influenza viruses were resistant to Ose [6]. Soon after the pandemic was declared, the emergence of the first Ose resistant A(H1N1)pdm viruses was reported [7]–[9].

There is substantial evidence to support that the emergence of mutations associated to phenotypic resistance to Ose are bound to become fixated independently of Ose-induced selective pressure [10], [11], as a result of complex genetic interactions involving selection at multiple levels, including host interactions [12]. Thus, adequate screening is of great importance for the identification of novel variants associated with clinically and epidemiologically relevant phenotypes.

Genetic analysis of influenza virus involves partial or complete genome sequencing which is costly and time consuming. Current methods for the identification of Ose resistance are based on the identification of mutations in the NA gene by sequencing or by measuring enzymatic activity of NA in the presence of the NA inhibitors, which requires not only viral culture, but for biosafety requirements is not feasible on a large scale [5]. Thus, epidemiological monitoring of the genetic diversity of influenza virus and Ose resistance and other clinically relevant phenotypes require new methods for screening large numbers of individuals [13].

NGS has revolutionized the field of genomic research. Due to low cost per base and enormous throughput, it has been used to study the genetic diversity in different contexts such as microbial diversity [14], [15]. Metagenomic analysis using unbiased NGS has led to pathogen discovery, and has recently been used to characterize samples of patients with seasonal and pandemic influenza [16]–[18]. The “ultra-deep” sequencing approach involves sequencing thousands to millions of copies of a gene segment which is assumed to be genetically diverse. In the field of infectious diseases, “ultra-deep” sequencing was successfully used in the identification and quantification of low frequency genetic variants (or quasi-species) in individuals infected with HIV and hepatitis virus (HBV and HCV), as well as to estimate the impact of viral quasi-species in response to anti-viral treatment [19]–[22]. NGS was recently applied to the analysis of intra-host influenza virus diversity [17], [23], [24] and its dynamics upon oseltamivir treatment [25].

Hereby, we investigated whether for epidemiological surveillance of viral genetic diversity NGS could be used to sequence viral samples from a large number of individuals at a lower intra-individual depth, in contrast to the “ultra-deep” sequencing of low numbers of samples to a great depth. To test this, we conducted a population-based study to screen the genetic diversity of Influenza A(H1N1)pdm virus in patients of the State of Morelos, Mexico by massive sequencing of a 265 bp NA gene segment during the period May to November, 2009.

## Results

### Epidemiological Analysis

**Figure 1:**
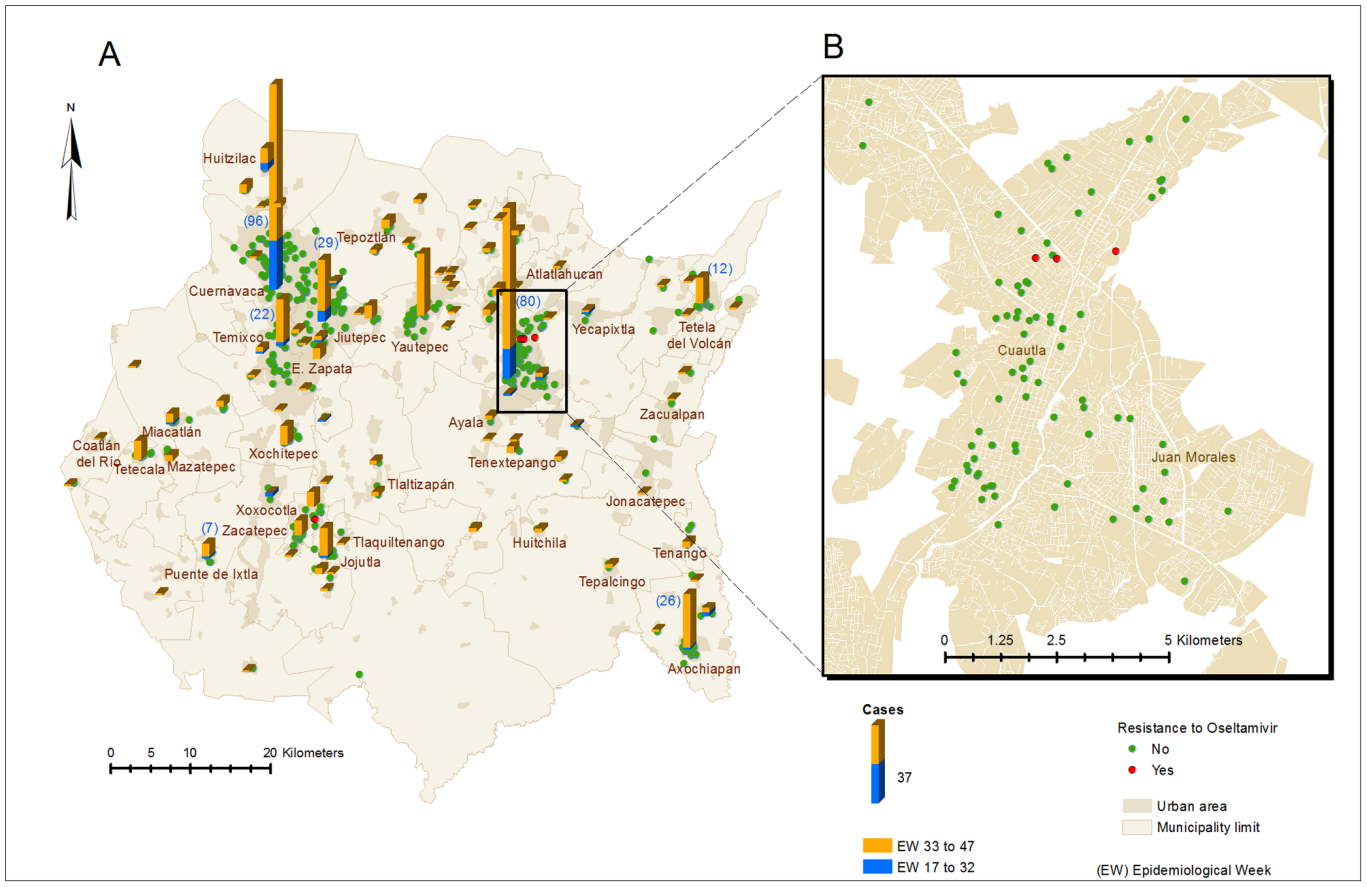
Distribution of A(H1N1)pdm influenza cases during May-Nov, 2009 period. **A)** Overview of the State of Morelos, Mexico. Bars represent the number of cases according to municipality during the first wave (weeks 17–32; blue section of each bar) and during the second wave (weeks 33–47; orange section of each bar). Each individual case is represented in green (haplotypes not associated with oseltamivir resistance) and in red (either the H275Y or the S247N mutations). **B)** Zoom in of Cuautla city where three clustered cases of the S247N mutation were found.

Socio-demographic and clinical characteristics of our study population are shown in Table 1 and Figure 1A. To investigate if the 299 samples included in the sequencing process were representative of influenza cases reported in the State of Morelos during the period of the study, we compared epidemiological variables of our study population to all 3,196 patients reported as influenza-like illness at the state level during the study period. Our sample had a greater proportion of cases in the 2 to 20-year age range (162/299 (54.2%) versus 1141/3196 (35.7%), p  =  <.001); had a higher probability of living in the northwest (130/299 (43.5%) versus 952/3098 (30.7%), p  =  0.0035) and were less likely to live in the northeast state regions (122/299 (40.8%) versus 1745/3098 (56.3%), p  =  0.0009). Other demographic and epidemiological variables showed no significant differences. The comparison of our sample group with the sub-group of 786 virologically confirmed influenza cases revealed no significant differences. There was also no statistical difference between the 299 cases included in the study and the 513 cases diagnosed at the National Institute for Public Health (INSP) or the sub-sample that could not be amplified by RT-PCR (214 cases) (Table 1).

**Table 1:** Socio-demographic indicators, symptoms and co-morbidities of the sampled population.

Socio-demographical indicators	Total	Amplification failure	Successfully amplified	P*
	n/total (%)	n/total (%)	n/total (%)	
Female	240/506 (47.4)	100/207 (48.3)	140/299 (46.8)	0.742
0 a <2	19/506 (3.8)	4/207 (1.9)	15/299 (5.0)	0.183
2 a <20	266/506 (52.6)	104/207 (50.2)	162/299 (54.2)	0.183
20 a <45	174/506 (34.4)	75/207 (36.2)	99/299 (33.1)	0.183
45 a 65	44/506 (8.7)	23/207 (11.1)	21/299 (7.0)	0.183
≥ 65	3/506 (0.6)	1/207 (0.5)	2/299 (0.7)	0.183
Northwest	84/207 (40.58)	123/299 (41.14)	207/506 (40.91)	0.751
Northeast	76/207 (36.71)	122/299 (40.8)	198/506 (39.13)	0.751
Southwest	19/207 (9.18)	23/299 (7.69)	42/506 (8.3)	0.751
Southeast	22/207 (10.63)	24/299 (8.03)	46/506 (9.09)	0.751
Foreigner	6/207 (2.9)	7/299 (2.34)	13/506 (2.57)	0.751
**Clinical symptoms**				
Fever	505/506 (99.8)	207/207 (100.0)	298/299 (99.7)	0.405
Headache	504/506 (99.6)	207/207 (100.0)	297/299 (99.3)	0.238
Rhinorrhea	497/506 (98.2)	205/207 (99.0)	292/299 (97.7)	0.25
Dyspnea	461/506 (91.1)	191/207 (92.3)	270/299 (90.3)	0.444
Polypnea	90/506 (17.8)	46/207 (22.2)	44/299 (14.7)	0.03
Malaise	456/506 (90.1)	187/207 (90.3)	269/299 (90.0)	0.89
Cough	493/506 (97.4)	205/207 (99.0)	288/299 (96.3)	0.058
Conjunctivitis	253/506 (50.0)	101/207 (48.8)	152/299 (50.8)	0.651
Myalgia	456/506 (90.1)	188/207 (90.8)	268/299 (89.6)	0.659
Odynophagia	378/506 (74.7)	158/207 (76.3)	220/299 (73.6)	0.484
**Co-morbidities**				
Allergy	4/506 (0.79)	0/207 (0.0)	4/299 (1.34)	0.095
Asthma	15/506 (3.0)	5/207 (2.4)	10/299 (3.3)	0.545
Chronic obstructive pulmonary disease	5/506 (1.0)	0/207 (0.0)	5/299 (1.7)	0.062
Diabetes	13/506 (2.6)	6/207 (2.9)	7/299 (2.3)	0.697
Hypertension	2/506 (0.4)	1/207 (0.5)	1/299 (0.3)	0.793
Obesity	3/506 (0.6)	1/207 (0.5)	2/299 (0.7)	0.789
HIV positive	2/506 (0.4)	1/207 (0.5)	1/299 (0.3)	0.793
Smoker	12/506 (2.4)	8/207 (3.9)	4/299 (1.3)	0.066

* ***X^2^*** test.

### Pooled Libraries Sequencing

As mentioned in the previous section, of 513 initial laboratory confirmed cases, we were able to obtain RT-PCR amplification products of 299 samples which were pooled in 48 libraries representing between 5–7 subjects clustered in time and space. The failure to amplify viral cDNA from the other samples can be attributed to a combination of lower viral loads, variation in sample handling prior freezing, variation in freezing-thawing cycles and prolonged storage (approximately 6–8 months) since the nasopharyngeal swabs were frozen at −80°C until processed for the present study. However, as shown in Table 1, no significant socio-demographical features were found between the amplified samples versus those that failed to amplify.

The pyrosequencing run of the 299 amplicons pooled in the 48 libraries yielded 80,422 sequences (one fourth of an optimal run). Sequencing metrics are detailed in Table 2. Using the Amplicon Variant Analyzer (AVA) software 42,599 reads were mapped to the reference amplicon (NA sequence of the A/Mexico/4487/2009 (H1N1); GenBank accession number: FJ998214.1) and belonged to one of the 48 libraries (i.e. were barcoded). The average read number per region was 5,325, with an average of 885 reads per library. We estimated an average of 144 reads per amplicon (individual) by dividing the number of reads per barcoded library by the number of individual amplicons in each (Table 2).

**Table 2:** Overall metrics and results of the sequencing run.

Process	Indicator		SD	Range
**Input**	Total of individuals	299	Na	Na
	Total number of pooled amplicon libraries	48	Na	Na
	Average number pooled amplicons per library	6.2	0.5	5–7
**Sequencing run**	Total number of reads	80,422	Na	Na
	Average number of reads per region	9,156	± 4231	5,724–18,037
	Number of sequenced bases (Mbp)	13.03	Na	Na
	Average read length (bp)	162	± 82	42–362
	Median read length (bp)	217	Na	Na
**Library analysis**	Mapped reads (usable reads)	42,599	Na	Na
	Average number of reads per region	5,325	± 3150	1,117–10,878
	Average number of reads per library	885	± 560	112–2,754
	Expected number of reads per individual	142	± 93	18–442
	Average depth at pos.823 per library	834	±582	79–2,746
	Expected depth at pos.823 per individual	139	±97	13–364
	Proportion of reads per barcoded library	0.167	± 0.05	0.063–0.283
	Estimated overall proportion of reads per individual library	0.036	± 0.018	0.009–0.053
**Control reads**	Number of control reads	1,303	Na	Na
	Average number of control reads per region	160.9	±79.8	25–270
	Average proportion of control reads per region	0.017	±0.009	0.004–0.029
	Overall per base accuracy	0.995	±0.005	0.958–0.999
	Average per base accuracy per region	0.996	±0.004	0.994–0.998

Na. Not applicable.

To assess sequencing error, we included a control amplicon which has the same sequence composition to the wild-type virus except for a stop codon at positions 823–825. This control amplicon lacked barcode and was spiked at a 1∶100 ratio on a molar basis with the rest of the libraries. We identified 1,303 control reads, with an average of 161 reads per region which corresponded to an average of 1.7% of the total sequencing yield per region (Table 2). Average sequencing accuracy of the control amplicon was 0.995 and average accuracy per region was 0.996, which is the expected accuracy for the 454 sequencing platform [26].

**Figure 2:**
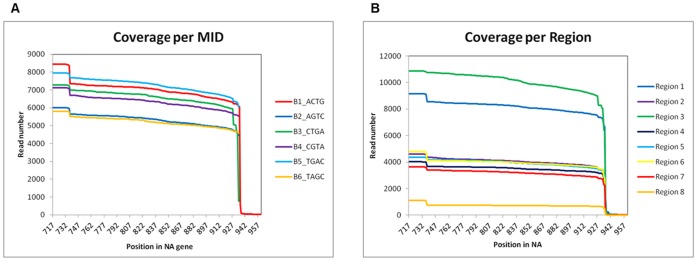
Sequence coverage plots of the sequenced NA amplicon according to barcode (A) and according to picotiterplate region (B). Positions of the NA gene are shown in X axis, whereas the number of reads is shown in Y axis. Less variability in read number was obtained according to MID than according to sequencing region, suggesting that the source of variability is during the emPCR amplification.

In addition to sequencing accuracy, reliable identification of genetic variants is dependent on adequate sequencing depth and amplicon length coverage. We observed that for all libraries, amplicon length was covered uniformly from NA positions 717 to 935 (Figure 2). There was less variation in sequencing depth according to each of the six barcodes used (average 7,100 ± 1,042 reads per barcode) (Table 2 and Figure 2A), than according to sequencing picotiter plate region (average 5,325 ± 3,151) (Table 2 and Figure 2B). Sequencing yield was particularly low in region 8 (Figure 2B). The high variation of sequencing yield among sequencing regions was likely to be due to increased proportion of unmapped short reads derived from primer dimer contamination, as well as non-clonal amplification during the emulsion PCR step, thus compromising the sequencing yield for some sequencing regions. The high accuracy per region (Table 2) indicates that even in regions where sequencing depth is low (i.e, region 8), accuracy remained high (not shown).

By using six barcoded pooled libraries (each containing 5–7 pooled amplicons with the same barcode) per sequenced region, the proportion of reads of each barcoded library is expected to be 0.166. The standard deviation of the proportion of reads per barcode per region was ± 0.05 and was used as proxy to measure pooling errors due to inaccuracies in amplicon DNA quantitation and liquid handling.

### Analysis of Genetic Diversity

**Figure 3:**
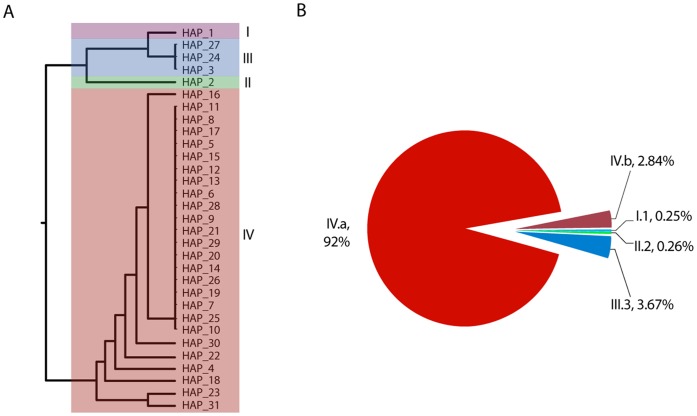
Genetic diversity and frequency of genetic variants identified in the 717–982 region of the NA gene of Influenza A(H1N1)pdm virus. **A)** Phylogenetic tree based on the average distance of the percent identity between translated sequences from the 30 identified haplotypes. The group I and II represent unique haplotypes with non-synonymous changes. Group III contains the reference haplotype (InDRE 4487) and two synonymous variants. Group IV contains the D248N mutation, seven non-synonymous and 19 synonymous variants. **B)** Frequency of haplotype variations in the total sample according to the major genetic groups, calculated from the number of sequencing reads for each haplotype or genetic group.

The comparison of the sequences in each of the 48 libraries with the NA gene reference sequence of InDRE (A/Mexico/4487/2009 (H1N1); GenBank accession number: FJ998214.1) identified 27 single nucleotide mutations with a frequency greater than 1%. This threshold is within the range of previously published work using the 454 platform [20], [21], [27], as well for the fact that using the AVA software, reads from the control amplicon spiked at a 0.4–2.9% generated a single haplotype. Of the 27 identified variants, 17 were synonymous and 10 non-synonymous (Table 3). A detailed analysis of AVA clustered sequences revealed that the 27 mutations comprise 30 new haplotypes with a frequency greater than 1% within each pooled library (Table 3, Figure 3A). The 30 haplotypes were classified according to their sequence in four founder groups (I–IV), including the reference haplotype (III). As shown in Table 3, the groups I and II contained a single haplotype each. Besides the reference haplotype, group III contained two additional synonymous haplotypes (III.24 and III.27) at the protein level. Group IV was highly predominant and contains the remaining 26 haplotypes (Figure 3B). Group IV is defined by the non-synonymous change 742: A/G (N248D). In this group, 19 haplotypes have additional synonymous changes (Group IVa) and seven involve one or more non-synonymous changes in addition to N248D (Group IVb) (Table 3, Figure 3A).

**Table 3:** Influenza A (H1N1)pdm NA gene variants identified.

Haplotype	Variant	AA	Group	Sub-group	Global frequency (%)	Estimated number of individuals&	Validation by Sanger sequencing
I	740:G/T,786:G/A	S247I,K	I	NA	0.25	1	Yes
II	746:G/A	G249E	II	NA	0.26	1	Yes
III.a.3	InDRE 4487	NA	III	NA	2.85	9	Yes
IV.b.4	742:A/G,807:G/A	N248D, M269I	IV	b	0.46	1	Yes
IV.a.5	742:A/G	N248D	IV	a	84.12	251	Yes
IV.a.6	732:T/C,742:A/G	D,N248D	IV	a	0.37	1	
IV.a.7	742:A/G,906:G/A	N248D, P	IV	a	0.24	1	
IV.a.8	742:A/G,921:C/T	N248D, N	IV	a	0.02	0	
IV.a.9	742:A/G,930:G/A	N248D, L	IV	a	0.7	2	
IV.a.10	742:A/G,747:A/G	N248D, G	IV	a	0.15	1	
IV.a.11	742:A/G,828:T/C	N248D, Y	IV	a	0.07	0	
IV.a.12	742:A/G,804:A/G	N248D, E	IV	a	0.78	2	
IV.a.13	742:A/G,768:C/T	N248D, F	IV	a	0.11	1	
IV.a.14	742:A/G,765:C/T	N248D, I	IV	a	0.38	1	
IV.a.15	742:A/G,822:T/C	N248D, Y	IV	a	0.42	1	
IV.b.16	742:A/G,823:C/T	N248D, ***H275Y***	IV	b	0.21	1	Yes
IV.a.17	742:A/G,759:C/T	N248D, Y	IV	a	0.39	1	
IV.b.18	742:A/G,790:G/A	N248D, V264I	IV	b	0.16	1	
IV.a.19	742:A/G,753:C/T	N248D, A	IV	a	0.2	1	
IV.a.20	742:A/G,783:A/G	N248D, G	IV	a	0.38	1	
IV.a.21	742:A/G,894:C/T	N248D, G	IV	a	0.3	1	Yes
IV.b.22	742:A/G,922:C/A	N248D, Q308K	IV	b	0.28	1	Yes
IV.b.23	740:G/A,742:A/G	***S247N***, N248D	IV	b	1.14	3	Yes
III.a.24	900:T/C	N	III	NA	0.18	1	
IV.a.25	742:A/G,897:G/A	N248D, S	IV	a	3.48	12	
IV.a.26	742:A/G,747:A/G,897:G/A	N248D, G, S	IV	a	0.44	1	Yes
III.a.27	762:G/A	K	III	NA	0.64	2	
IV.a.28	742:A/G,762:G/A	N248D, K	IV	a	0.39	1	
IV.a.29	741–742:TA/CG	S,N248D	IV	a	0.03	0	
IV.b.30	741–742:TA/CG,860:A/G	S,N248D,E287G	IV	b	0.02	0	
IV.b.31	740:G/T,742:A/G	S247I,N248D	IV	b	0.58	2	

Bold italics: Mutations associated with oseltamivir resistance.

& Rounded quotient of the proportional frequency of the haplotype within each barcoded library divided by the actual number (corrected) of amplicon in each barcoded library.

**Figure 4:**
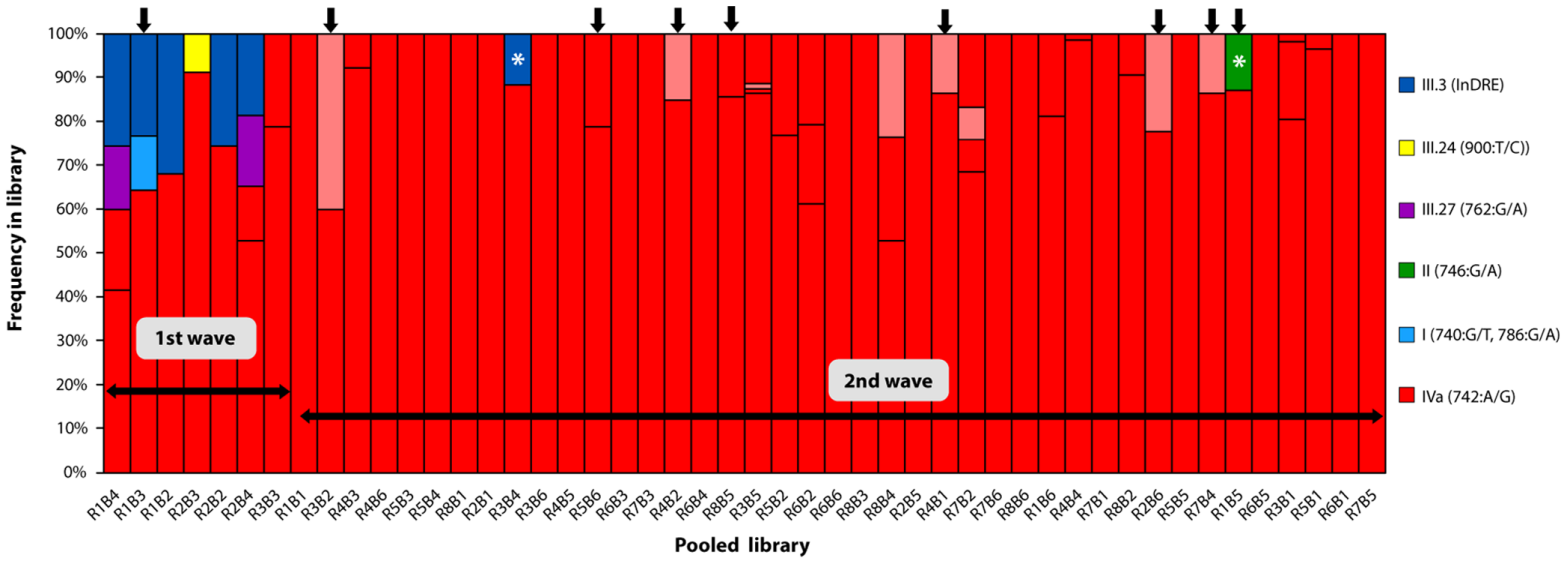
Genetic diversity of Influenza A(H1N1)pdm virus in the State of Morelos (2009). The graph shows the proportion (Y axis) of each of the four genetic groups described in **Table 3** in each of the 48 libraries, (X axis) ranked according to epidemiological week of the latest individual in the pool. Thus, the earliest cases are seen in the left, whereas the later cases are shown at right. The red dots represent the haplotypes of group IVb, i.e. those haplotypes that contained additional non-synonymous mutations to D248N. Black arrows in the top represent libraries in which individual amplicons were validated by Sanger sequencing. The asterisk (*) indicates those libraries which included an individual from the first wave.

The approximate frequency of different haplotypes was analyzed by calculating the global proportion of reads for each haplotype in the whole sequencing run (Table 3, Figure 3B) or in each library (Figure 4). This analysis showed a marked predominance of haplotypes of group IV (95.8%). Group III haplotypes, the reference (InDRE 4487) and III.24 and III.27 synonymous haplotypes (3.6%) followed, and the remaining corresponded to groups I and II, present in only one library each, suggesting that the original virus was quickly replaced by variants corresponding to group IV (Figure 3B) (see the following section: Viral genetic variation dynamics during 2009).

An important limitation of our experimental design is that it does not reveal the identity of the 5, 6 or 7 samples included in each library. However, we wondered if a reliable estimation of absolute frequency could be estimated (i.e, number of individuals having each of the 31 identified haplotypes). Assuming that equimolar amplicon quantification and pooling were performed accurately, depending on the number of amplicons in each library, it would be expected that between 14.5, 16.6 and 20% of the sequences in each library were from a single individual (see “Proportion of reads per barcoded library per region” in Table 2). We assumed that barcoded library pooling error (± 0.05) is the same as individual library pooling error, and that pooling error is independent of sequencing yield according to picotiterplate region. By simply calculating the normalized proportions of the 48 libraries (Figure 3B) divided by the average proportion corresponding to a single individual (0.1618), we inferred that the number of individuals for variants I, II, III, IVa and IVb was 1, 1, 11, 274 and 8, respectively. The calculated sum of individuals (295) was lower than the actual number of individuals (299). This is likely to be because the number of individuals per library was variable (3, 31 and 9 libraries included five, six and seven individuals, respectively). To avoid this problem, we also estimated the number of individuals with each haplotype by dividing the proportional frequency of the haplotype within each barcoded library by the corrected denominator according to the actual number of individuals in each pooled library followed by rounding. However, the estimated number of individuals having variants I, II, III, IVa and IVb was 1, 1, 12, 279 and 12, respectively and the sum of individuals (302) was higher than the actual number of individuals sequenced. The approximate frequency of each haplotypes calculated by the later approach is detailed in Table 3.

To confirm the existence of single-individual haplotypes, Sanger sequencing of individual amplicons corresponding to nine pooled libraries (R1B3, R1B5, R2B6, R4B1, R8B5, R3B2, R4B2, R5B6 and R7B4) was performed (Table 3 and Figure 4). For R1B3, R1B5, R5B6 libraries, all amplicons were sequenced and the presence of haplotypes I, II, IVb.16 and IVa.26 in a single individual was confirmed (see section “Identification of genetic variants associated with oseltamivir resistance” and Table 3). For pools R2B6, R4B1 and R8B5, we were able to sequence only 4/6, 5/6, and 4/6, respectively. Nevertheless, this was enough to confirm the existence of IVb.4, IVb.22 and IVa.21 in one of the sequenced amplicons of the respective libraries. The occurrence of other haplotypes that occur in more than one individual such as III, IVb.5, IV.b23 was also documented within these pools at the predicted frequency (see section “Identification of genetic variants associated with oseltamivir resistance” and Table 3). Overall, Sanger sequence validation was performed in 51 individual amplicons which correspond to 17% of the total number of amplicons sequenced by NGS.

### Viral genetic Variation Dynamics during 2009

In order to understand the dynamics of viral diversity with respect to the initial evolution of the pandemic, we analyzed the frequency distribution of the different genetic variants identified with respect to the library of origin. As mentioned in Methods, the libraries were built with "pools" of amplicons from individuals related by time (week) and space (municipality of residence); however, in some cases this grouping was not all that strict. The libraries were ranked according to epidemiological week of the case that was diagnosed last in each cluster as a proxy for a temporary order. As shown in Figure 4, the early phase of the epidemic (weeks 14–28, the “first wave”) was characterized by a mixed pattern of groups I, II, III and IVa, although with a predominance of IVa (over 60%). As the pandemic progressed, the presence of haplotypes I, II and III was replaced by variants of group IV that completely dominated the “second wave” (*p* < 0.001, X^2^). Although haplotypes III.3 and II were found in the second wave (R3B4 and R1B5 libraries), it is likely that they correspond to one of the individuals in the first wave that were sub-optimally pooled during library generation. In the case of the R1B5 library (haplotype II), this assumption was confirmed by Sanger sequencing of each amplicon in the library (Figure 4). Derived from the temporal analysis, our results from pooled amplicon libraries suggest that the original variants described in April 2009 were quickly displaced by the haplotypes of group IV. The emergence of non-synonymous variants of group IV (IVb) was observed only during the second wave, although we cannot assert that such variants arose exclusively during this period.

**Figure 5:**
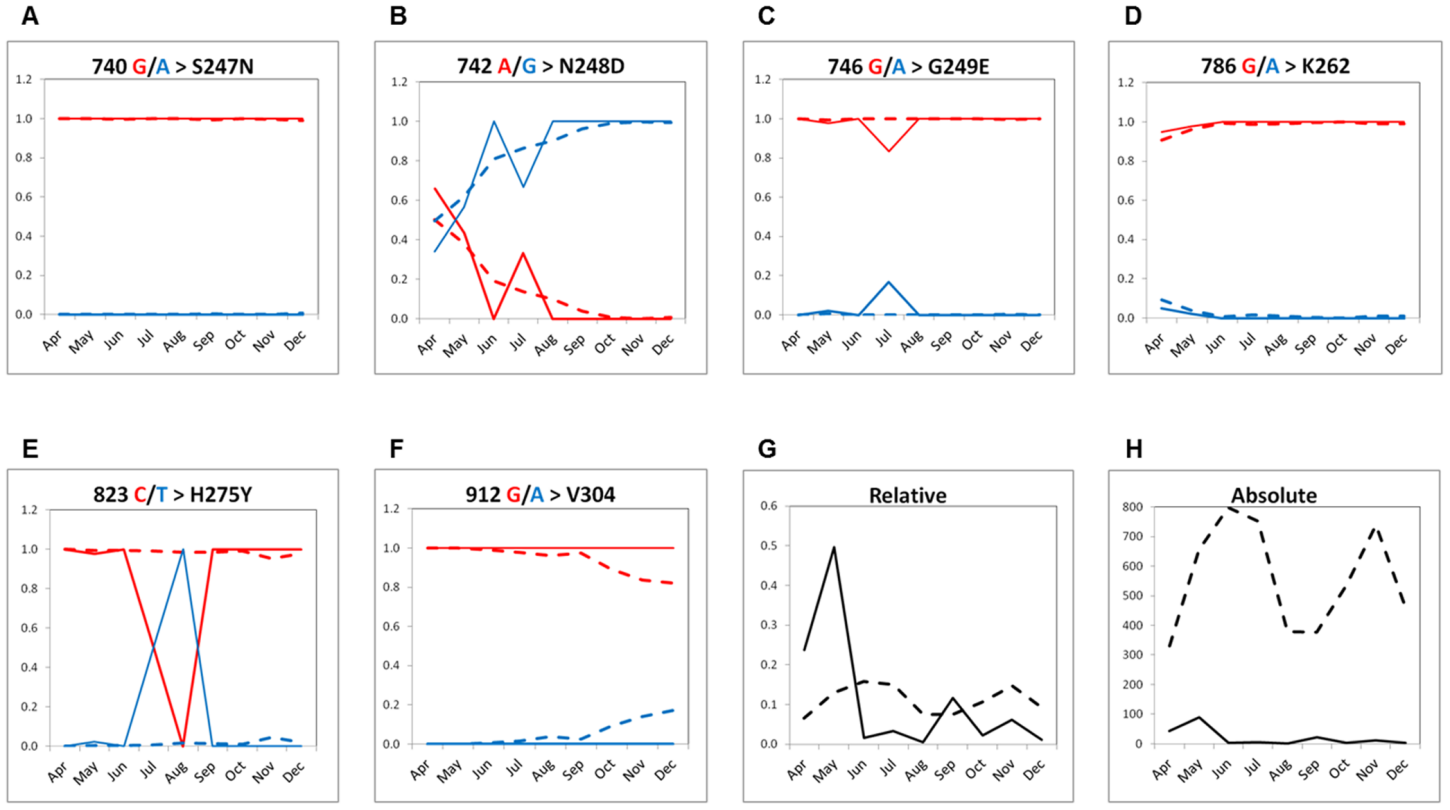
Analysis of the relative frequency of Influenza A(H1N1)pdm virus NA mutations identified in this study (A–E) that were reported in the GenBank in 2009 (April to December) in Mexico (n  =  181) and the rest of the world (n  =  5,032). Dotted lines represent world-wide isolates, whereas solid lines represent Mexican isolates. Red and blue lines represent alternate alleles. The 912 G/A mutation was not found neither in Morelos nor in Mexican GenBank reports, but was identified elsewhere (**F**). The absolute (**G**) and relative (**H**) number of isolates used for this analysis is shown for Mexico (solid black line) and world-wide isolates (dotted black line). Note that in June, July and August, only 3, 6 and 1 Mexican isolates were reported in the IVR, respectively.

We further analyzed viral genetic diversity dynamics by data mining of NA influenza sequences reported in the Influenza Virus Resource (IVR) (http://www.ncbi.nlm.nih.gov/genomes/ FLU/FLU.html) during the April-December, 2009 period. Neuraminidase sequences covering the full amplicon from 181 Mexican and 5,032 non-Mexican isolates were retrieved. The relative frequency of 740 G/A, 742A/G, 746 G/A, 786 G/A, 823C/T alleles was estimated for all isolates (Figure 5). The 921 G/A allele not found in Mexico was also included for comparison. The 742A allele (group III) was highly prevalent during the first pandemic wave and was quickly replaced by 742G (group IV), both in Mexico and the rest of the world (Figure 5B) [28]–[30]. Similarly, the 786A allele (group I) was observed at a rather low frequency during April and May, 2009, but was absolutely replaced by the 786G allele in the upcoming months (Figure 5D). The 740G allele was very stable during both pandemic waves (Figure 5A). The 746A allele was seen only during the first wave (two cases in May and one in July), but was absent in the second wave (Figure 5C). Of note, six 823C/T Mexican isolates (associated to oseltamivir resistance) were found (Figure 5E). Of these, two (GenBank accessions CY064412 and CY062542) were isolated since May, 2009. Finally, the 922G/A allele was not observed in Mexico in our sequencing effort nor in GenBank reports. However, the relative frequency of 922G appeared to decline and showed a tendency for replacement by the 922A allele at the end of 2009 (Figure 5F).

Overall, our results describing the genetic diversity of the pandemic virus derived of pooled library sequencing of samples from the State of Morelos are consistent with those sequences reported in the IVR from Mexican and non-Mexican isolates [28]–[30].

NGS has been widely used in the genetic analysis of viral variants at intra-individual level (viral quasi-species) [19]–[22]. As discussed previously, we estimate that between 14.5 and 20% of sequencing reads from each library correspond to one individual. As shown in Table 3, haplotype frequencies for IVa.8, IVa.11, IVa.29 and IVb.30 were less than 4%, (i.e. less than one third of the expected ratio for a single viral type per individual). The number of reads for the IVa.8, IVa.11, IVa.29 and IVb.30 haplotypes was 8, 27, 26 and 22, respectively. Thus, it is possible that these variants could represent viral quasi-species, however due to the inherent sequencing error rate, the low sequence coverage and its variability within pooled libraries (Figure 2), this cannot be ascertained.

### Identification of Genetic Variants Associated with Oseltamivir Resistance

**Figure 6:**
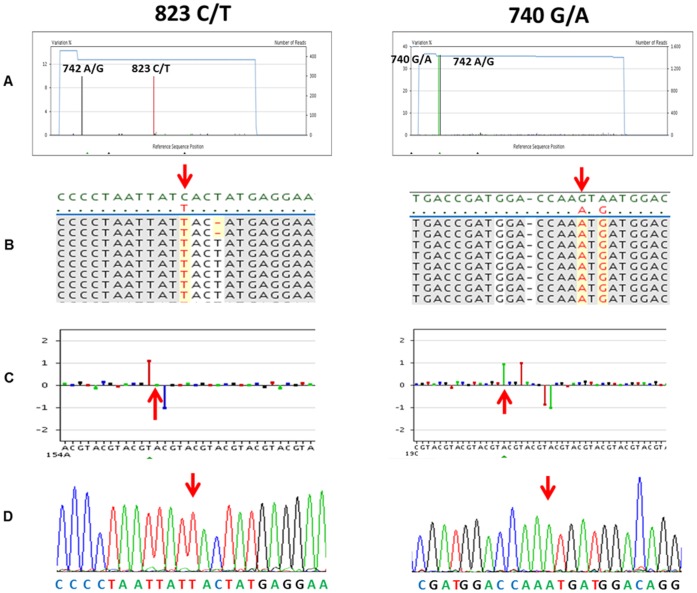
Identification by massive sequencing of the IVb.16 (left panel) and IVb.23 (right panel) mutations associated to oseltamivir resistance. **A**) View of haplotype IVb.16 and haplotype IVb.23 consensus alignment showing the frequency for each individual variant for either haplotype: A/G (black bar) and 823 C/T (red bar) for the H275Y mutation; 740 G/A (green bar) and 742 A/G (black bar) for the S247N mutation. **B**) Detail of aligned reads. The reference sequence (InDRE 4487) is shown in green. The mutations 823 C/T (left panel) and both 740 G/A and 742 A/G (left panel) are shown in red with a yellow background. **C**) Representative pyrosequencing flowgrams showing luminous intensity difference at position 823 (left panel), and 740 and 742 (right panel) with respect to the reference (InDRE 4487). **D**) Sanger sequencing chromatograms corresponding to individuals #791 and #281 belonging to libraries R7B4 (left panel) and R3B2 (right panel) representing the confirmation of genotypic resistance conferred by the H275N and S247N mutations, respectively.

The haplotype IVb.16 corresponds to genotypes 742: A/G, 823: C/T, which determines the changes N248D and H275Y, respectively. H275Y mutation is associated with resistance to Ose in the pandemic virus as well [31], and was identified only in one library (R7B4). The mutation represented 9.92% of the sequences obtained in this library (Figure 4). As mentioned previously, to determine if the H275Y mutation corresponded to a single patient of the corresponding pool or minority variants from different individuals from the same pool, each individual amplicon contained in library R7B4 was subjected to Sanger sequencing. These sequences confirmed that the H275Y mutation accounts for one sample obtained from a 35 year-old women from the city of Zacatepec, Morelos, whose sample was taken during week 42 and received oseltamivir treatment before obtaining the sample (Figure 1; Figure 6, left panels).

Haplotype IVb.23 corresponds to the genotypes 740:G/A and 742:A/G, which code for the S247N and N248D non-synonymous changes, respectively. The S247N mutation involves the NA active site and has been described to confer resistance to Ose [8]. Haplotype IV.23 was present in libraries R3B2 and R4B2 at a frequency of 40 and 14%, respectively (Figure 4). All the cases in both libraries were from the City of Cuautla, Morelos during week 38 to 40. As for haplotype IV.16, 12 individual amplicons belonging to such pools were also subjected to Sanger sequencing allowing the confirmation of two cases aged 14 and 16 years old in library R3B2 (week 38) and an additional 2-year old boy in library R4B2 (week 39). None of them had received oseltamivir treatment and lived within a 1.5 kilometer distance (Figure 1B; Figure 6, right panels).

## Discussion

We present here a proof of principle that shifting the "ultra-deep" sequencing approach (aimed at identifying minority viral variants) [17], [32] towards the inclusion of large numbers of individuals (at the expense of lowering sequencing depth) can be applied to the genetic monitoring of influenza virus during epidemics. In this approach, low sequencing depth (an estimation of 142 ± 93 reads per individual) was sufficient for confident variant consensus assignment, which is determinant of the maximum number of viral samples to be sequenced. The implementation of this strategy on a population based study provided temporal and spatial description of relevant viral characteristics.

Within the context of the 2009 A(H1N1)pdm influenza, the epidemiological situation of the State of Morelos described in this work was no different from that described in population-based studies in other parts of Mexico and the rest of the world [33], indicating that the proposed strategy can be generalized. In the short period analyzed in this study (May-November, 2009), we found 30 virus variants. The analysis of the genetic variant dynamics showed that the reference haplotype (III.a.3) isolated in April 14th, 2009 and published in GenBank in May 13^th^ (FJ998214.1), as well as haplotypes I and II that were observed during the first pandemic wave were replaced by variants belonging to group IV, containing the mutational signature 742G/A (N248D), which dominated during the second wave (Figure 4). This observation is consistent with the prevalence and dynamics of such mutations in Mexican and non-Mexican isolates reported in the IVR (Figure 5) and has been described previously for world-wide isolates [28], [29], [30]. The high prevalence of the N248 allele observed in April in Mexico (66%) (Figure 5B) suggests that this was one of the most basal allele and is consistent with studies of genetic diversity during the first wave of the pandemic [28]. Nevertheless, the interphase between the first and second pandemic wave seems to represent a population bottleneck where the initial genetic variants containing N248 belonging to haplotypes I, II and III (clades 1–3 [28], cluster 1 [29]) were displaced by variant D248 during the second wave. Whether this replacement was adaptive or resulted from genetic drift remains to be elucidated, however studies of HA and NA diversity in isolates obtained early in the 2009 pandemic have shown that the N248D substitution segregates with an S220T substitution in the HA gene [28]–[30], which may be the result of complex co-selective processes such as compensatory mutations involving binding affinity for sialic acid mediated by HA and virion release mediated by NA [12], [34].

The great viral diversity found in the State of Morelos and the infrequent geographical clustering is consistent with recent high resolution studies of viral dynamics despite substantially different methodological approaches [35]. Although limited to a short amplicon, the proposed surveillance strategy allowed the characterization of viral dynamics across time; more importantly, genetic variants of the virus associated with clinically relevant phenotypes such as resistance to Ose conferred by the H275Y mutation in one case, and the S247N mutation in three additional cases. The finding of three cases harboring this mutation in the same location suggests frequent transmission of this viral haplotype. The first published report of a H275Y mutation in Mexico documented a case that occurred in January, 2010 [36], few months after the one reported here (November, 2009). Intriguingly, data mining of retrieved NA sequences from the IVR revealed that the H275Y mutation appeared in Mexico since May, 2009. Such findings suggest that the frequency of the H275Y mutation may be underestimated. The prevalence of the S247N mutation has been low throughout the world; however its frequency raised in the Asian Pacific region during 2010–2011 [8]. The low number of Mexican isolates publicly available at the IVR was striking (Figure 5H), taking into account the scientific, social and political impact of the pandemic in Mexico. This may be the result of insufficient genetic surveillance, limitations in the release of sequence data to public repositories or both.

The surveillance approach presented here is proposed as an adjuvant for rapid, reliable and extensive routine monitoring in developing countries, where infrastructure and trained human resources are centralized and limited. However, under a translational research point of view and aiming to field applicability and eventual application in viral phylogenomic analysis, the ideal system for genetic screening would require: 1) high-throughput to allow large numbers of samples to be sequenced; 2) longer read length to allow for better resolution of genetic diversity and eventually full viral genome sequencing; 3) capacity to identify each sample included in the sequencing process; 4) labor-friendly and quick protocols for library preparation; and 5) low cost per sample.

Regarding throughput and read length, we used the 454-Roche GS FLX platform, which can generate 3.5–4 x 10^5^ reads of up to 250 bp in length in a single sequencing run. The yield was approximately one fourth of the maximal expected yield, mainly due to an excess of short reads that are discarded during the signal processing and mapping to the reference amplicon. The cause of short reads may be attributable to primer dimer contamination of the pooled libraries, which can be corrected by size purification. The 454-Roche GS FLX Titanium platform yields 8 x 10^5^ reads with a median length of 450 bp, thus allowing great number of samples to be screened. It also allows the screening of other positions involved NA inhibitors resistance such as the Q136K and I223V-R [3], [5] by increasing the sequenced region span. Other sequencing platforms that have been used for “ultra-deep” sequencing such as Illumina [32], could also be used with the proposed approach because they have several-fold increased throughput, and coupled with multiplexing could increase proportionally the number of samples. However, shorter read lengths would limit the possibility of screening a larger gene region. Whereas higher throughput increases the accuracy and sensitivity of minority variant identification, larger read lengths enable the detection of new variants with unexpected phenotypic effects and facilitate haplotype reconstruction [37]. In a near future, higher throughput and long reads will merge in a single platform, which in combination with full length viral genome sequencing [38] and the approach provided here may provide an extensive capacity for influenza surveillance suitable for deep understanding of population and evolutionary virology analysis.

The proposed strategy has the limitation of not allowing the immediate identification of every sampled individual because it relies on amplicon pooling to increase the number of tested individual amplicons. It should be emphasized that to avoid false representation of viral haplotypes, pooling has to be performed equimolarly at the RT-PCR amplicon level, as was done in the present study, not at the RNA or nasal swab sample. Nevertheless, errors in the correct estimation of haplotype frequencies could arise from inaccurate amplicon quantification and liquid handling error. We postulate that amplicon pooling can be useful to increase sample numbers and reduce costs, as long as the samples in the pool are related temporally and geographically. In this way, the identification of a significant genotype (i.e. Ose resistance) allows targeting interventions to a temporally and geographically defined population of individuals that make up the original pool. The criteria for pooling samples can be defined by the research team according to the nature of the outbreak and can be as wide (i.e. municipality, district, state) or narrow (i.e. blocks, schools, hospitals) as necessary. In this work, criteria for defining sample pooling was done manually, based exclusively on the epidemiological week and municipality of origin, but we could not guarantee that the optimal array was achieved. If this strategy is scaled up, manual clustering could represent a challenge and developing clustering algorithms would be required. If the ultimate identification of each sample is required, barcoding could be increased. In this work we used a very limited number of barcodes (six). However, the number of validated barcodes for sequencing has been increasing allowing the routine use of 96-plexed samples [39]. Other approaches such as paired-barcoding [40] or the combinatorial pooling strategy or DNA Sudoku can be harnessed to allow individual sample identification [41]. Taken together, increased sequencing throughput and the ability of increasing the multiplexing factor, we consider feasible to sequence 3,120 samples with an average of 1,500 reads per pool (250 reads per individual) with an array of 520 libraries (pools of six individuals per library) classified by 65 barcodes in an eight-region plate in a single 454-Roche GS FLX Titanium run.

Rapid and ease sample preparation is desired for epidemiological surveillance in order to guide intervention strategies. Library preparation is time consuming and requires highly trained personnel in molecular biology. In the present work, all the process was performed manually, but increasing sample number would require the use of liquid handling robots [42], [43]. Recent advances in microfluidics allow rapid, simple and large scale preparation of barcoded libraries at low cost [44].

The cost of high-throughput sequencing has been dropping dramatically. A 454-Roche GS FLX sequencing run costs about 10,000 USD. In this work, the estimated cost to detect one positive pool was 200 USD, and an additional 60 USD to identify individual samples of the corresponding pool by Sanger sequencing, which is still very high. However, considering that the results presented here derived from a sub-optimal yield sequencing run (one fourth of its capacity), together with a yield increase in the 454-Roche GS FLX Titanium platform, we can anticipate the possibility to cost-efficiently implement this approach. In this scenario, with a sequencing run of 3,120 samples, such as the one mentioned above, the cost to genetically characterize a single pool would be close to 20 USD. The sequencing run cost of other NGS technologies such as Illumina and Ion Torrent are substantially lower at the expense of read length. Altogether, the concomitant reduction in cost per base, increased read length, multiplexing and the high-throughput generation of barcoded libraries will allow the implementation of schemes for sequencing thousands of isolates at affordable costs in a near future.

A conceptually similar approach to ours termed “ultra-wide” sequencing has been recently published in the context of HIV antiviral resistance genotyping [45]. The main difference is that Dudley *et al*, used the 454 GS junior bench top sequencer which enable them to sequence 48 barcoded non-pooled samples. The strategy described here can be used to study many problems related to pathogen genetic diversity from a global health perspective and can be added to the growing number of applications of NGS to virology research [46]. In the particular case of influenza, it can be used to track drug resistant variants and genetic variation dynamics, as exemplified by our study. Other interesting applications can lead to epidemiological scale monitoring of virus antigenic variation, which is essential for the selection of the strains to be incorporated in the annual seasonal vaccine preparations. This strategy can also be used to monitor transmission of other species of influenza virus, which may pose a threat to global health, e.g. avian influenza. Recently, it has been described that few mutations in the hemagglutinin gene of H5N1 avian influenza virus are sufficient for airborne transmission from human to human [47], [48]. The approach presented here could be useful in risk assessment of airborne human to human transmission by screening H5N1 avian influenza by massive screening of circulating viruses in birds, pigs and humans.

## Materials and Methods

### Ethics Statement

Nasopharyngeal swab specimens analyzed in this study were collected by the National Institute of Public Health (INSP) as part of the Epidemiological Surveillance Program conducted by the Morelos State Health Services between the months of May to November, 2009, thus no informed consent either, written or oral was obtained. The remains of each sample were stored in a sample bank, and its use for this study was approved by the National Institute of Public Health Ethics committee. Accordingly, the origin of samples and personal information were handled anonymously.

### Sample Selection and Epidemiological Analysis

Nasopharyngeal swab samples were stored at -80 °C until use. Confirmation of Influenza A(H1N1)pdm virus infection was performed according to the real-time WHO PCR protocol reported in April, 2009, (http://www.who.int/csr/resources/publications/swineflu/realtimeptpcr/en/index.html).

The state of Morelos is located in the center of the Mexican Republic, has an extension of 4,892.73 km2 and has approximately 1,777,227 inhabitants, 16.1% of whom live in rural communities (fewer than 2,500 inhabitants) (INEGI. http://www.inegi.org.mx/sistemas/mexicocifras/default.aspx. Consulted in July, 2012). During the period mentioned above, 3,150 cases of influenza-like illness were reported throughout the state to the State Epidemiological Surveillance Program, of which 786 (24.5%) were confirmed as Influenza A(H1N1)pdm virus infections. Of these, 513 were diagnosed at INSP, and 299 cases, on which we were able to obtain PCR amplification products, were included in this study.

General epidemiological data for each clinical sample included in the study was recovered from the National System for Influenza Surveillance (SISVEFLU) as a part of the National Epidemiological Surveillance Division of the National Ministry of Health. Differences between socio-demographical variables in patients with influenza-like illness (n  =  3,150), the total state confirmed cases (n  =  786), the subsample diagnosed at INSP (n  =  513) and our study population (n  =  299) were examined using a binomial test.

### Viral RNA Extraction and Generation of Amplicons

Viral RNA was purified from 140 µl of each nasopharyngeal swab specimen using the QIAamp Viral RNA mini kit (QIAGEN) following the manufacturer's protocol. The viral RNA was used for individual amplification of an internal 265 bp fragment of the NA gene (positions 717 - 982) of the NA sequence of the A/Mexico/4487/2009 (H1N1) isolate as reference (GenBank accession number: FJ998214.1). The amplicon was 307 bp long, including fusion primers and allowed near to full length sequencing using the 454-Roche Genome Sequencer FLX platform (average read length 250 bp), covering position 823 associated to the H275Y mutation (CAC to TAC substitution). Gene specific primers were designed as fusion primers with adaptors A and B according to the GS FLX Amplicon Library Preparation Method. To allow library multiplexing in the same sequencing run, different sets of fusion primers were used, including one of six possible 4 bp barcodes (multiple identifiers or MID’s) (Table 4).

**Table 4:** Oligonucleotide sequences used in this study.

Oligonucleotide	Sequence 5′ – 3′	Use
OsemutF	5**′**-AATTAT**TAA**TATGAGGAATG-3**′** *	Mutagenesis
OsemutR	5**′**-TCCTCATA**TTA**ATAATTAGG-3′*	Mutagenesis
AoseF	5′-AdaptAFlx {454 key}TGTAAATGGTTCTTGCTT-3′	Control library
BoseR	5′-AdaptBFlx {454 key}TGCCTGTCTTATCATTAG-3′	Control library
BoseF1	5′-AdaptBFlx {454 key}***ACTG***TGTAAATGGTTCTTGCTT-3′	Barcoded library 1
BoseF2	5′-AdaptBFlx {454 key}***AGTC***TGTAAATGGTTCTTGCTT-3′	Barcoded library 2
BoseF3	5′-AdaptBFlx {454 key}***CTGA***TGTAAATGGTTCTTGCTT-3′	Barcoded library 3
BoseF4	5′-AdaptBFlx {454 key}***CGTA***TGTAAATGGTTCTTGCTT-3′	Barcoded library 4
BoseF5	5′-AdaptBFlx {454 key}***TGAC***TGTAAATGGTTCTTGCTT-3′	Barcoded library 5
BoseF6	5′-AdaptBFlx {454 key}***TAGC***TGTAAATGGTTCTTGCTT-3′	Barcoded library 6
AdaptAFlx	5′-AGCCTTGCCAGCCCGC-3′	Sanger sequencing
AdaptBFlx	5′-GCCTCCCTCGCGCCA-3′	Sanger sequencing

* Underlined nucleotides indicate the stop codon for mutagenesis in positions 823–825.

AdaptAFlx refers to the 454 adaptor A sequence. AdaptAFlx refers to the 454 adaptor B sequence. 454 key indicates 454 library key sequence (TCAG). Nucleotides in bold italics indicate barcodes. Regular font indicates NA gene specific sequence (699–716; 983–1000).

The sequencing amplicons were generated by end-point RT-PCR using SuperScript III One-Step RT-PCR System with Platinum Taq High Fidelity (Invitrogen) in 25 µl reaction volume. Reaction conditions were the following: 1 cycle at 42 °C for 60 minutes, followed by one cycle at 94 °C for 3 minutes and 45 cycles of denaturation for 30 seconds at 94 °C, alignment at 60 °C for 30 seconds and extension for 30 seconds at 68 °C and a cycle final extension for 5 minutes at 68 °C. Amplification products were examined on 1.5% agarose gels and purified in Wizard columns according to the manufacturer's protocol (Promega), and quantified using Nanodrop.

To estimate the error rate during amplicon generation and massive pyrosequencing, an identical NA control amplicon was generated without barcodes and with a stop mutation (codon CAC to TAA at positions 823–825) that codes for an H275 stop substitution. This would yield a non-functional NA which is unlikely to derive from functional viral progeny. This mutation was generated by site-directed mutagenesis using mutagenic oligonucleotides OsemutR and OsemutF, according to the protocol described [49] (Table 4). The mutation was confirmed by Sanger sequencing.

### Generating and 454 Sequencing of Pooled NA Amplicon Libraries

To avoid erroneous representation of viral variants within each barcoded pool due to unequal amount of virus titers in each individual sample, the 299 amplicons were mixed in equimolar amounts (100 ng of ds cDNA of each amplicon per pool) in 48 pools of five to seven amplicons, prior to the sequencing process. Amplicons in the same pool shared the same barcode, thus reads from the five to seven individuals could not be identified. However, we attempted to include in the same pool samples from individuals clustered in the same municipality and in the same epidemiological week, although this was not possible for all pools. Using this approach, the 48 barcoded libraries containing five to seven unidentifiable individual amplicon samples related in time and space were constructed. For sequencing, the 48 libraries were divided in eight groups of six differentially barcoded libraries and were subjected to emulsion PCR (emPCR). The control amplicon (mutated version of NA) was emulsified with each group of libraries in a molar ratio of 1∶100. After emPCR breaking, sequencing was carried out in a single LR70 picotiter plate separated with an eight region gasket on the 454-Roche Genome Sequencer FLX platform, according to the manufacturer's instructions.

### Sequence Analysis

Sequences were analyzed using the 454-Roche GS Amplicon Variant Analyzer 2.6 version. This program allows each library to be analyzed independently and according to the assigned barcode. Control library reads were identified by the lack of barcode sequence. The sequences generated were compared with the NA sequence of the A/Mexico/4487/2009 (H1N1) isolate as reference (GenBank accession number: FJ998214.1), which was one of the first isolates reported at the beginning of the pandemic (isolated in April 14^th^, 2009; published in GenBank in May 13^th^, 2009). The AVA software was used to identify novel haplotypes and to estimate the relative amounts in each library, based on read number belonging to each haplotype. To account for possible sequencing errors [26], [50], we defined a true haplotype as the consensus sequence containing at least 1% of all reads present in a given library [20], [21], [27], and because AVA generated a single consensus in control reads spiked at a ∼1% proportion. Once identified, haplotype variants were compared with those present in the IVR (http://www.ncbi.nlm.nih.gov/genomes/FLU/FLU.html) using local BLAST and Linux Shell Programming.

For position based coverage analysis and estimation of sequencing accuracy of control amplicons, mapped reads aligned to reference amplicon were extracted from the AVA project and transformed to a tab-delimited matrices. Estimation of coverage depth and accuracy (average proportion of correct matches per position regarding the Sanger sequenced control amplicon) was performed with a custom R script.

### Sanger Sequencing

The RT-PCR products from 25 individuals belonging to R3B2, R4B2, R4B6 and R7B4 libraries were sequenced directly by the Sanger method using primers AdaptAFlx and AdaptBFlx with a 3100 DNA Genetic Analyzer, Applied Biosystems (Table 4). The chromatograms were visualized using Chromas 2.33 version and compared manually.
